# Comparison of whole brain radiation therapy for synchronous brain metastases with irradiation protecting the hippocampus versus whole brain radiotherapy for sequential brain metastases to boost irradiation in the treatment of brain metastases from SCLC: study protocol for a randomized controlled trial

**DOI:** 10.1186/s13063-022-06826-4

**Published:** 2022-10-14

**Authors:** Xiaofang Zhang, Tianlu Wang, Chen Yu Wang, Peng Zhao, Bo Huang, Lei He, Ying Qiu Song

**Affiliations:** 1 Department of Radiotherapy, Cancer Hospital of China Medical University, Liaoning Cancer Hospital and Institute, No.44 Xiaoheyan Road, Dadong District, Shenyang, 110042 Liaoning Province People’s Republic of China; 2 Department of Radiotherapy Physics, Cancer Hospital of China Medical University, Liaoning Cancer Hospital and Institute, No.44 Xiaoheyan Road, Dadong District, Shenyang, 110042 Liaoning Province People’s Republic of China

**Keywords:** Brain metastases from SCLC, Hippocampus protection, TOMO, Intracranial progression-free survival time, Assessment of neurological function

## Abstract

**Background:**

This study is in regard to the comparison of whole brain radiation therapy for synchronous brain metastases with irradiation protecting the hippocampus versus whole brain radiotherapy for sequential brain metastases to boost irradiation in the treatment of brain metastases from small cell lung cancer (SCLC). Therapeutically, they have notably varying dose distributions. Based on theoretical and model studies, it has long been speculated that these modes may result in different prognostic outcomes. We aim to assess the efficacy of tomotherapy in the treatment of SCLC brain metastases while protecting the key functional area, the hippocampus, and minimizing any neurocognitive impairments incurred by radiation.

**Methods:**

This is a randomized, controlled, prospective study including 102 SCLC patients with brain metastases randomized (1:1) to the experimental (whole brain radiation therapy for synchronous brain metastases with irradiation to protect the hippocampus) or control (whole brain radiotherapy for sequential brain metastases to boost irradiation) group. The sample size is calculated through a single-sided test; 102 participants will be required for the main results to have statistical and clinical significance. We aim to provide clinical trial data support for better prognostic treatment options in patients with SCLC and brain metastases. The clinical trial data include both the primary and secondary outcomes; the primary outcome is the intracranial progression-free survival time after the new technology application. The secondary study outcomes include the assessment of neurological function, the quality of life, and the overall survival rate. Follow-up consultations will be conducted every 2 months. After the final patient completes follow-up, the Statistical Product and Service Solutions software will be used for scientific and rigorous data analysis. Version 1.0 of the protocol was implemented on January 1, 2021; the recruitment process for this clinical trial commenced on April 1, 2021, and will end on March 31, 2024.

**Discussion:**

The study will provide high-quality clinical evidence to support the efficacy and safety of whole brain radiation therapy for synchronous brain metastases with dose irradiation protecting the hippocampus versus whole brain radiotherapy for sequential brain metastases with push volume irradiation for the treatment of patients who have lung cancer as well as brain metastases. This has not been previously reported.

**Trial registration:**

This trial is registered with the Chinese Clinical Trial Registry (ChiCTR1900027539; November 17, 2019) (URL: https://www.chictr.org.cn/hvshowproject.aspx?id=20515).

## Background

Approximately 80% of small cell lung cancer (SCLC) patients develop brain metastases during the entire course of the disease [1, 2]. The craniocerebral brain is one of the common metastatic sites for SCLC and is mainly treated by radiation therapy, which can significantly prolong the survival time of patients [3]. Whole brain radiotherapy (WBRT) is a basic radiation therapy specially employed in patients with extensive brain metastases [4–6]; however, it is associated with side effects including neurotoxicity and cognitive dysfunction. Radiation-induced hippocampal damage plays an important role in cognitive dysfunction; thus, avoiding the hippocampus can effectively reduce the cognitive impairment attributed to WBRT [6–9].

Studies have shown that the metastasis rate of SCLC in the hippocampus is approximately 3–5.1% [10–13]. Modern radiotherapy modalities include conventional three-dimensional conformal radiation therapy (3D-CRT) and intensity-modulated radiation therapy (IMRT) [14]. Spiral tomotherapy (TOMO) is another novel method in the field of IMRT technologies that uses a spiral 360° radiation transmission system, similar to that of spiral computed tomography (CT) scanning. Most planning studies have demonstrated that IMRT can fully treat the target volume while retaining healthy organs and tissues, thereby outperforming 3D-CRT [15–17]. Compared with traditional fixed-field IMRT, TOMO possesses the advantage of the use of more independent ray directions, which could result in better dose adaptation to the target. By quickly opening and closing the blades in a collimator rotating around the patient, TOMO can maintain the radiation dose in a tumor area of complex shape, while preventing exposure of normal, healthy organs to radiation [18, 19]. Compared with 3D-CRT, TOMO can reduce the number of tissues irradiated at a dose level equivalent to the tumor dose and redistribute the excess dose to normal tissues, thereby resulting in a total integrated dose that is smaller than that required by 3D-CRT [20, 21].

Currently, TOMO is often used for a variety of diseases [22–24]; however, the clinical value of TOMO in the treatment of SCLC brain metastasis remains controversial. There is a substantial difference regarding the dose distributions of the abovementioned technologies. Based on theoretical and model studies, it has been long speculated that different doses may result in different prognostic outcomes. Therefore, this study sought to use TOMO technology to implement WBRT synchronous irradiation of brain metastases with increased radiation doses, while protecting the key functional areas of the hippocampus.

Our primary hypothesis is that TOMO will increase the clinical intracranial progression-free survival (iPFS) time by approximately 5–8%, owing to the higher accuracy of the treatment plan regarding location and dose of local tumor control. Our secondary hypothesis is that neurological function will be better protected due to the accuracy of this therapy. In the future, we hope to develop intervention methods and improve the corresponding treatment protocols based on the results of this clinical trial. This clinical trial is expected to further improve the efficacy of radiotherapy and improve the quality of life and survival of patients.

## Methods/design

### Study settings

This clinical trial will be conducted at the Liaoning Cancer Hospital Medical Center (a tertiary care center) in China. This is a prospective study enrolling 102 patients. We aim to investigate the safety and efficacy of radiotherapy in patients with brain metastases caused by SCLC using the TOMO treatment device. Using the random number table method, a random number sequence will be generated, and patients will be assigned to the experimental and control groups in a 1:1 ratio. The experimental group will use TOMO, while the control group will use three-dimensional conformal radiotherapy (conventional 3D-CRT)/intensity-modulated radiation therapy tomotherapy (IMRT). This study will not use blinding of trial participants since the treatment procedure clearly distinguishes between the trial and control groups.

The trial sponsors are the Shenyang Major Scientific Research Projects [No. 191124090] and the Cancer Research Program of National Cancer Center [NCC2017A08]. This is version 1.0 of the protocol as of January 1, 2021. Recruitment for this clinical trial began on April 1, 2021 and will end on March 31, 2024.

### Study participants

Liaoning Cancer Hospital is a tertiary specialty hospital in Liaoning Province, China, with a large number of oncology radiotherapy patients each year. The patient’s treating physician explains this clinical trial to patients who meet the enrollment criteria and obtains the patient’s consent to achieve sufficient participant enrollment. A computer will automatically generate the allocation sequence using the random number table method. Team investigators will recruit participants and assign them to the intervention. Assignments will be implemented using sequentially numbered, opaque, and sealed envelopes that are unknown to the investigator who contacts the participant prior to assigning the intervention. Since the trials are all clinical treatments, compliance is good, and the experimental progress can be viewed according to the archive of treatment cases in the computer.

### Eligibility criteria

#### Inclusion criteria

1. Satisfying the diagnostic criteria for conventional 3D-CRT/IMRT and TOMO
2. An age between 18 and 70 years
3. A KPS score of > 80 points at the baseline visit
4. Presence of SCLC brain metastasis, with ≤ 5 brain metastases
5. No brain stem or bulbar metastasis
6. No important organ failure
7. Agreement to participate and sign an informed consent form

#### Exclusion criteria

1. Severe damage to important organs such as the heart, liver, kidney, etc.
2. Uncontrolled, severe systemic infection, sepsis, or toxicemia
3. Hemoglobin < 80 g/L or white blood cells < 3.0 × 10^9^/L before treatment
4. Advanced cancer with anemia, weight loss, or cachexia
5. Having participated in other clinical studies

### Sample size

It will be necessary to evaluate differences between the two groups; thus, the sample sizes of the experimental and control groups were set as n1 and n2, respectively. The two groups should have the same sample sizes; therefore, a single-sided test was used. The sample size calculation formula was:

$${n}_1={n}_2=2{\left[\frac{\left({z}_{\alpha }+{z}_{\beta}\right)\sigma }{\delta}\right]}^2$$

where σ is the overall standard deviation (estimated to be 1.84); δ, the difference between the two sets of numerical variables (estimated to be 1); Zα, the standard normal value corresponding to the inspection level α; and Zβ, the standard normal value corresponding to β. If *σ* = 1.8 months, *δ* = 1 month, *α* = 0.05, *β* = 0.20, Zα/2 = Z0.05/2 = 1.96, and Zβ =Z0.20 = 0.842; then, by substituting the above formula, we obtain

$${n}_1={n}_2=2{\left[\frac{\left(1.96+0.842\right)\times 1.8}{1}\right]}^2=50.9\approx 51$$

Hence, 51 cases will be included in the control group and 51 cases in the experimental group, with the total sample size being 102 cases.

### Primary study outcome

#### Intracranial progression-free survival

The primary study outcome is iPFS, defined as the number of months from the start of TOMO or conventional 3D-CRT/IMRT to the occurrence of the first evidence of disease progression, which will be evaluated according to the Response Evaluation Criteria in Solid Tumors.

### Secondary study outcomes

#### Assessment of neurological function

The Expanded Disability Status Scale (EDSS) assessment will be used to standardize the neurological examination, and each system will be scored according to the degree of dysfunction.

#### Quality of life

Quality of life (QoL) will be assessed using the European Organization for the Research and Treatment of Cancer Quality of Life Questionnaire (EORTC QLQ-C30) (version 3) in patients with brain metastases caused by SCLC at the baseline and every 2 months after treatment. The questionnaire is available as an attachment to the paper.

#### Overall survival

Overall survival (OS) refers to the number of months between the radiotherapy procedure and the time of death or last follow-up. For patients who are lost to follow-up prior to death, the time of the last follow-up visit is usually calculated as the time of death.

### Study outline

TOMO—a collection of current advanced radiotherapy concepts—is a new, image-guided IMRT system that uses CT scanning to emit radiation in a fan-shaped spiral. The application of novel technologies, such as spiral irradiation, image guidance technology, and adaptive radiation therapy, improves the accuracy of radiotherapy and reduces the radiation damage incurred to normal tissues.

#### Equipment and technical support

A three-dimensional treatment planning system (3D-TPS), treatment plan system (TPS), 3D water tank, linear electron accelerators, image fusion software, TOMO, medical linear accelerators, and CT have been provided by the radiotherapy department. Equipment and technical support details are presented in Table 1.

**Table 1:** Summary of the new technology project equipment

Device	Device model	Manufacturer	Factory number
3D-TPS	pinnacle3	Philips	20050628
TPS	Pinnacle Smart Enterprise	Philips	20030126
3D water tank	RFA-300	IBA	20021210
Linear electron accelerator	IX6117	Varian	20151217
Image fusion software	MIM	MIM Software	20151125
Linear electron accelerator	UNIQUE	Varian	20140122
TOMO	Tomotherapy HD	Accuracy	20151125
Medical linear accelerators	Clinac IX	Varian	20151203
CT	SOMATOM	Siemens	20140731

*3D-TPS* three-dimensional treatment planning system, *TPS* treatment plan system, *3D water tank* three-dimensional water tank scanning system, *CT* computed tomography, *TOMO* tomotherapy system

#### CT localization scanning method

The patient will lie supine on the special positioning neck and shoulder stand for radiotherapy, and a polymer low temperature-hydrolyzed head, neck, and shoulder thermoplastic body membrane will fix the position. Next, the laser will be positioned on the midline of the head and on both sides (using a special external positioning system). After marking the corresponding reference points on the neck and shoulder thermoplastic body membrane and body surface, the lead particles will be placed for marking. A spiral CT during continuous, calm breathing will scan the whole brain with a pitch of 1 mm, transmitting the data to the TPS via the network prior to performing two-dimensional MRI image acquisition, with layer thickness 1 mm. After the attenuation of the MRI image is corrected with the CT data, the image will be reconstructed using an iterative method.

#### Formulation and implementation of radiotherapy plans

A radiotherapy department chief physician and an experienced image diagnosis physician will outline the radiotherapy target area on the MRI and CT fusion images. The tumor area and gross tumor volume and its extroverted 0.5 cm will be used as the clinical target area clinical tumor volume (CTV). CTV and its extroverted 0.3 cm and tumor motion range will be used as the planning target volume (PTV). Additionally, important functional areas will be outlined, such as the hippocampus, inner ear, and patient lens, and a TOMO radiotherapy plan on the Pinnacle^3^ treatment planning system will be developed. A Varian’s high-energy electron linear accelerator 6-MVX line will be used, alongside tomographic intensity-modulated irradiation field irradiation.

#### Treatment plan

Patients who meet the inclusion criteria will be randomly divided into two groups; the experimental group will receive whole brain radiation therapy for synchronous brain metastases with dose irradiation protecting the hippocampus, while the control group will receive whole brain radiotherapy for sequential brain metastases to boost irradiation. The experimental group will receive a WBRT dose of 40 Gy/2 Gy/20f five times per week. Simultaneously, radiotherapy doses of 60 Gy/3 Gy/20f will be provided for brain metastases five times per week, protecting the hippocampus. Brain irradiation is generally conducted based on important functional areas such as the inner ear and crystal; radiotherapy will be completed in 4 weeks. The control group will deliver WBRT doses of 40 Gy/2 Gy/20f five times per week. In addition, we will use radiotherapy at 27 Gy/3 Gy/9f for sequential brain metastases. The biological equivalent dose (BED) for brain metastases in the experimental group will be 72 Gy. The BED for brain metastases in the control group alone will be a total of 73 Gy (WBRT doses of 40 Gy, brain metastases-sequential boost 33 Gy). Therefore, BED analysis of the experimental (BED: 72 Gy) and control (BED: 73 Gy) groups will be considered consistent, and the choice of prescribed dose will be in line with the clinical diagnosis and treatment norms. All patients will undergo neurological function assessment before treatment to assess the occurrence of any neurological functional changes.

**Fig. 1:**
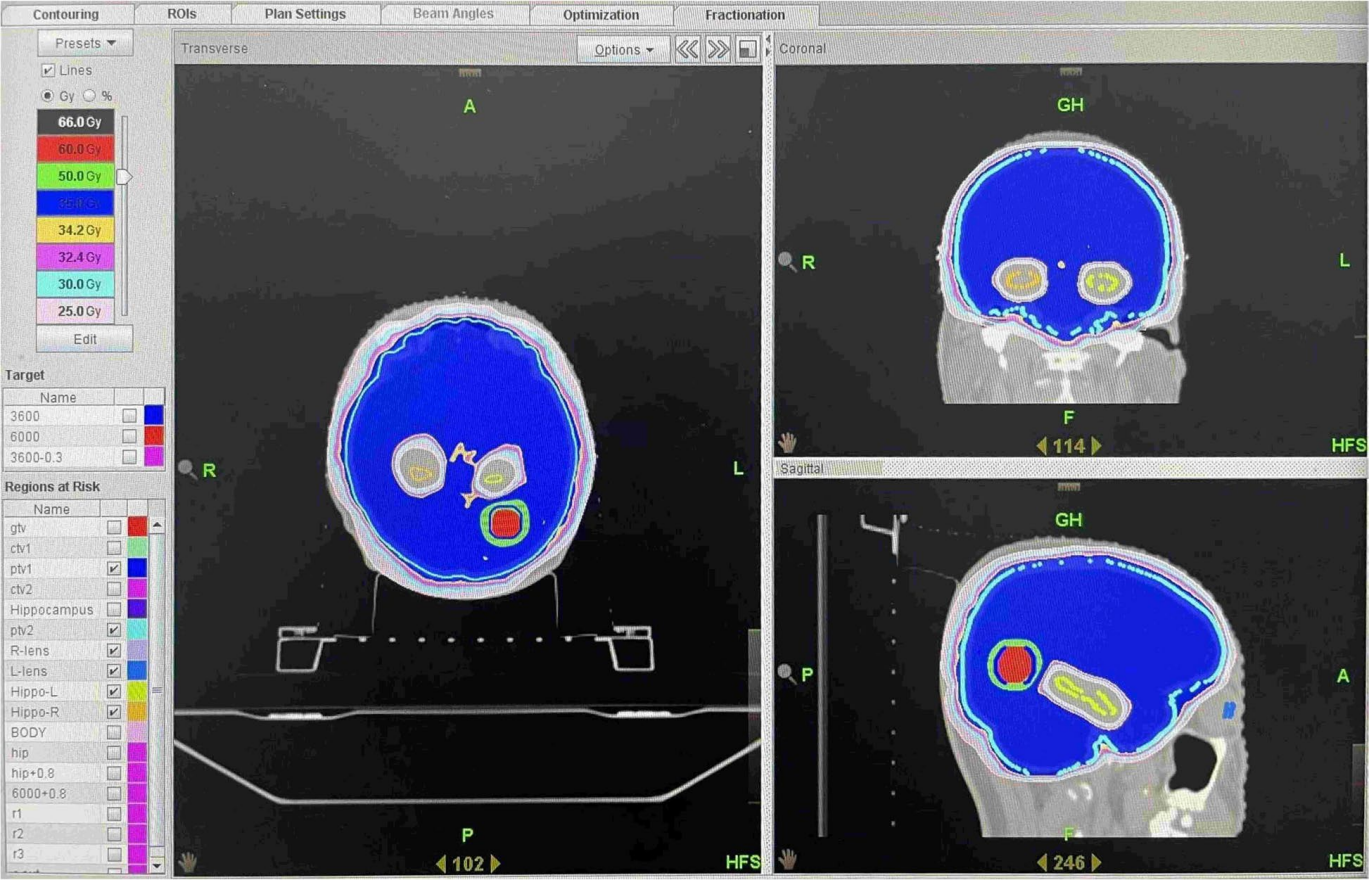
Contour of the hippocampal area and specific dose constraints

Hippocampal avoidance will occur in the control group; details on contouring of the hippocampal area and specific dose constraints are shown in Fig. 1.

#### Post TOMO treatment

Patients’ recovery will be monitored (both the experimental group and the control group) as per current medical practice. It may be necessary to perform additional medical treatments following these therapies if side effects appear. Patients undergoing the treatments can be discharged after a few days without any complications.

#### Post TOMO follow-up

**Fig. 2:**
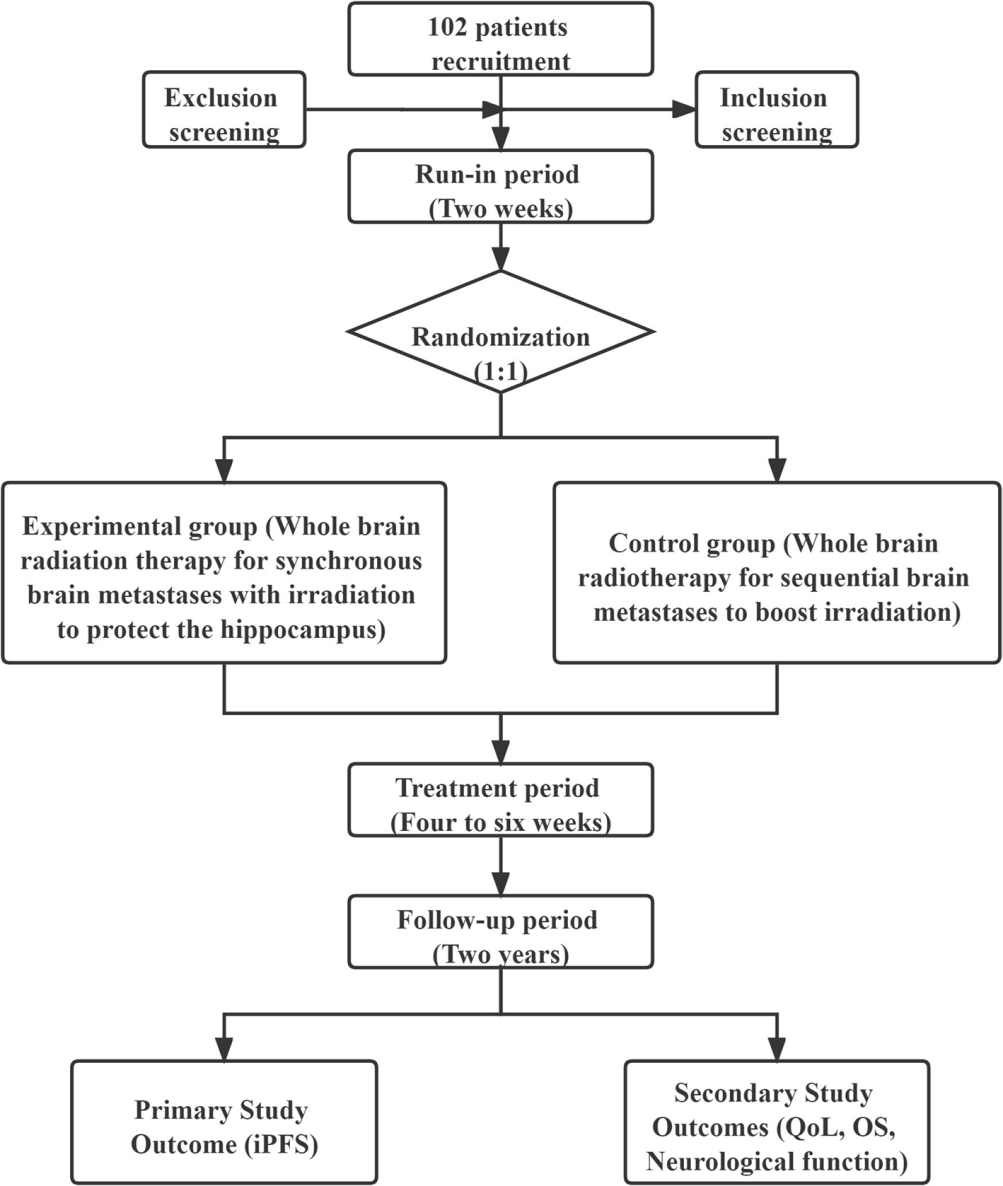
Flow chart representing the study procedures

After radiotherapy, patients will be reviewed every 2 months for a total of 6 months to evaluate whether they have recurrence and to determine their neurological function status. During 6 months to 2 years after treatment, a re-check will be performed every 6 months. The examination will include a brain MRI test, routine blood tests, and liver and kidney function tests, which will be used to evaluate the effectiveness of the therapy according to the standards set by the committee. The follow-up period for iPFS and OS is 2 years. In addition, we will assess the neurological function and QoL immediately post radiotherapy and at 1 month, 3 months, 6 months, 1 year, and 2 years post RT. The research outline is shown in Fig. 2 and Table 2.

**Table 2:** Summary of the research outline

	Study period						
	Run-in		Treatment				Follow-up
Time point	***-2W***	0W	***1W***	***2W***	***3W***	***4W***	***104W***
Visit	Visit1	Visit2	Visit3	Visit4	Visit5	Visit6	Visit7
**Enrollment**							
Eligibility screen	√	√					
Informed consent	√						
Demographics	√						
Randomization		√					
**Intervention**							
Experimental group							
Control group							
**Assessments**							
iPFS						**√**	**√**
Neurological function		√	√	√	√	√	√
QoL		√	√	√	√	√	√
OS						**√**	**√**

*W* week or weeks, *iPFS* intracranial progression-free survival, *QoL* quality of life, *OS* overall survival

### Data analysis

After the last patient completes the follow-up study, the committee will assess the occurrence of the primary and secondary endpoints. Treatments in participant timeline are all routine medical orders, and patients will follow the medical advice. If there is any failure to review on time, a phone call will be reminded. If the patient fails to complete the relevant examination, evaluation or follow-up within the agreed time, or cannot suspend the study, part of the clinical trial of the patient will be suspended. The data of these patients will be marked on the record and not entered in the final curative effect analysis system. The fixed staff is responsible for the input of data, coded into groups 1 and 2 according to their treatment plans, and set a password for the database to protect the privacy of patients’ data. Data quality is controlled by the Data Quality Supervision Committee.

Data will be organized and analyzed using the SPSS 25.0 software, and *P* < 0.05 will be considered significant in all statistical tests. iPFS and OS will be calculated using the Kaplan-Meier method. Assessment of neurological function will be scored using the EDSS assessment scale, and QoL will be scored using the FACT-G. The chi-squared test will be used in combination with propensity matching analysis to minimize differences due to smoking, sex, weight loss, geographic factors, economic status, and education level and to avoid baseline effects to the greatest extent possible. Definition of analysis population relating to protocol non-adherence is that the patients who do not follow. In addition, we use the multiple imputation method to handle missing data.

## Dissemination

### Data monitoring

The Data Monitoring Committee (DMC) is composed of all the authors, with Dr. Song Yingqiu as the team leader, responsible for testing the accuracy and authenticity of data and reviewing whether the operation of obtaining data is standardized. If the patient fails to complete the relevant examination, evaluation, or follow-up within the agreed time or cannot suspend the study, part of the clinical trial of the patient will be suspended.

### Adverse event report

A committee was formed for collecting, assessing, reporting, and managing solicited and spontaneously reported adverse events and other unintended effects of trial interventions or trial conduct. Summarize them to avoid recurrence to the greatest extent.

### Suspension of clinical trials standards

If a patient fails to suspend the clinical trial to complete the relevant tests, or to complete the follow-up assessment within the agreed upon time frame, in the following circumstances, or if the study if not suspended, that portion of the patient’s specific clinical trial will be suspended.

### Roles and responsibilities

The study sponsor and funders provide financial support in the research, and they will not have ultimate authority over any of these activities. The coordination center, steering committee, endpoint decision committee, and data management team are composed of all authors. Each item is a monthly regular meeting to monitor its quality. The coordination center is responsible for coordinating all resources, the steering committee guides the smooth progress of clinical trials, the endpoint adjudication committee is in charge of the ruling of endpoint events, and the data management team is responsible for monitoring the authenticity and quality of the data.

### Protocol amendments

The research group will have regular monthly meetings, and if there are revisions, they will be communicated.

### Auditing

Auditing trial conduct is conducted by the Data Monitoring Committee (DMC) every month. The procedure is to discuss and vote after the trial for each result.

### Confidentiality

When collecting, sharing, and maintaining personal information about potential and registered participants, all staff follow the principle of confidentiality and do not disclose or disseminate patient personal information.

### Ancillary and post-trial care

Compensation for those injured as a result of participating in the trial is based on hospital regulations. Give the corresponding amount of compensation. The clinical trial data will be analyzed and released as soon as possible.

### Dissemination policy

Before our team publishes the report, individual participants will not be able to publish patient data directly related to the trial. Our team will obtain the final dataset, form a writing committee, and publish the test results at regional and national conferences. The final result will be presented at a scientific conference and published in a peer-reviewed journal; this will be in accordance with the journal’s guidelines. Before our team publishes the report, individual participants cannot publish patient data directly related to the trial. Due to the confidentiality of patients’ personal information and privacy, the public is not allowed to access the complete agreement, participant-level datasets, and statistical codes.

## Discussion

Evidence from previous studies suggests that when compared with 3D-CRT, WBRT for brain metastases, dynamic IMRT, volumetric modulated arc therapy, and TOMO obtained satisfactory PTV brain coverage and the best homogeneity index [25]. However, compared with the control group, in the experimental group which used TOMO treatment for SCLC brain metastases, the prognostic impact on patients and QoL assessment—including neurological function—have not yet been reported. The results of this study will provide evidence for the efficacy and safety of this approach and may inform future trials of similar approaches in larger populations. Moreover, findings from this trial will play an important role in improving treatment options for patients with brain metastases from SCLC as well as in helping first-line physicians plan radiation therapy regimens for cancer patients. The main limitation of this study is the information bias caused by patient heterogeneity and selection bias, which could affect the usefulness of the data.

### Trial status

The recruitment of participants started on April 1, 2021, and is expected to continue until March 31, 2024.

On submission for publication, version 1.0 of the protocol is being used. 1 January 2021.

## Data Availability

The data of this study is available from the corresponding author upon reasonable request.

## Abbreviations

3D-CRT: Three-dimensional conformal radiation therapy
BED: Biological equivalent dose
CT: Computed tomography
CTV: Clinical tumor volume
EDSS: Expanded Disability Status Scale
IMRT: Intensity-modulated radiation therapy
iPFS: Intracranial progression-free survival
PTV: Planning target volume
QoL: Quality of life
SCLC: Small cell lung cancer
TOMO: Tomotherapy
WBRT: Whole brain radiotherapy
